# Technology Readiness Level Roadmap for Developing Innovative Herbal Medicinal Products

**DOI:** 10.3390/ph17060703

**Published:** 2024-05-29

**Authors:** Eduardo Pagani, Cristina Dislich Ropke, Cristiane Mota Soares, Sandra Aurora Chavez Perez, Paulo José Coelho Benevides, Barbara Sena Barbosa, Ana Cecilia Bezerra Carvalho, Maria Dutra Behrens

**Affiliations:** 1 Medical Department, Azidus Brasil, Valinhos 13271-130, SP, Brazil; 2 Centroflora Group, Innovation Department, Campinas 06460-040, SP, Brazil; 3 Project Management Office, Vice Direction of Education, Research and Innovation, Institute of Drug Technology Farmanguinhos, Oswaldo Cruz Foundation, Rio de Janeiro 21041-250, RJ, Brazil; 4 Business Office, Oswaldo Cruz Foundation, Campo Grande 79081-746, MS, Brazil; 5 GMESP, Brazilian Health Regulatory Agency, Agência Nacional de Vigilância Sanitária (ANVISA), Brasília 71205-050, DF, Brazil; 6 Natural Products Department, Vice Direction of Education, Research and Innovation, Institute of Drug Technology Farmanguinhos, Oswaldo Cruz Foundation, Rio de Janeiro 21041-250, RJ, Brazil

**Keywords:** phytotherapy, technology readiness level, herbal substance, herbal preparation, herbal medicinal products, drug discovery, drug development

## Abstract

Despite the vast global botanical diversity, the pharmaceutical development of herbal medicinal products (HMPs) remains underexploited. Of over 370,000 described plant species, only a few hundred are utilized in HMPs. Most of these have originated from traditional use, and only a minority come from megadiverse countries. Exploiting the pharmacological synergies of the hundreds of compounds found in poorly studied plant species may unlock new therapeutic possibilities, enhance megadiverse countries’ scientific and socio-economic development, and help conserve biodiversity. However, extensive constraints in the development process of HMPs pose significant barriers to transforming this unsatisfactory socio-economic landscape. This paper proposes a roadmap to overcome these challenges, based on the technology readiness levels (TRLs) introduced by NASA to assess the maturity of technologies. It aims to assist research entities, manufacturers, and funding agencies from megadiverse countries in the discovery, development, and global market authorization of innovative HMPs that comply with regulatory standards from ANVISA, EMA, and FDA, as well as WHO and ICH guidelines.

## 1. Introduction

Natural products (NPs) have historically been the primary source of lead compounds in drug discovery [1]. Living organisms have developed metabolites for hundreds of millions of years to improve their survival capacity, thus creating chemical diversity [2]. NP collections have increased chemical and steric complexity (e.g., more heterocycles and chiral centers) and display better drug-like properties than their synthetic counterparts, making them more useful for early-stage screenings [3,4,5]. More than 50% of all marketed therapeutic small molecules are either NPs, NP derivatives, or synthetic molecules whose pharmacophoric center was inspired by NPs, including some with traditional use [6,7]. 

Herbal preparations (HP) comprise hundreds of phytochemicals [8,9] that could address medical needs through multitarget mechanisms [10,11,12,13]. The possible synergy of multiple compounds, within a phytocomplex proposed decades ago, has been substantiated by advancements in systems biology and “-omics” [14].

Herbal medicinal products (HMPs) are a niche of NPs [15]. It is estimated that up to 80% of the world’s population relies on herbal medicines and medicinal plants as a primary source of healthcare [16], which can be beneficial in several non-communicable diseases [17]. The global herbal market was USD 60 billion in 2010 and is expected to reach USD 5 trillion by 2050 [18]. If properly managed, this market growth can generate income and convert local populations into conservation agents [19].

The world has currently 377,749 accepted plant species, mostly occurring in megadiverse countries [20]. Only 15% have been characterized phytochemically and 6% pharmacologically [21,22]. The number of species used in HMPs registered by the Food and Drug Administration (FDA), European Medicines Agency (EMA), and the Brazilian Health Regulatory Agency (ANVISA) is just a few hundred, and most like *Ginkgo biloba*, *Panax ginseng*, and *Echinacea purpurea* have been known for centuries. This occurs despite regulations that have existed for decades in Brazil, the EU, and the USA permitting the registration of innovative HMPs.

Among the 17 megadiverse countries, only two, namely the USA and China, have a consistent track record of pharmaceutical innovation. Encouraging the development of innovative HMPs originating from other countries could potentially stimulate their scientific and socio-economic progress, safeguard biodiversity, and introduce novel therapeutic options for medical needs [23,24,25].

The absence of innovation may partly stem from legal challenges. Sanitary regulations differ across countries [26,27], and there exist legal uncertainties in the operationalization of the Nagoya Protocol [28,29]. Brazil has addressed both issues. ANVISA has issued a regulatory framework for HMPs aligned with EMA [30]. The Brazilian Biodiversity Law [31] aligns with the Nagoya Protocol, offering legal certainty for the assessment of genetic resources and traditional knowledge, and benefit sharing [28]. Also, attempts by the European Community (EC) and the World Health Organization (WHO) provide models for multinational harmonization [32]. 

However, despite these advancements, innovations from Brazil and other biodiverse countries remain scarce, indicating that a significant factor may still be missing. The development of HMPs is a lengthy and intricate process that relies on the effective management of various capabilities and long-term funding. Furthermore, adaptations to previously planned tasks are often necessary. Given these challenges, we believe that a comprehensive yet adaptable roadmap outlining this process could assist research institutions, pharmaceutical companies, and funding bodies in establishing consistent, long-term partnerships aimed at fostering innovation.

NASA proposed the technology readiness levels (TRLs) decades ago to guide managers in assessing the aeronautical technology’s readiness and risks at specific development key points [33,34]. The concept has spread over many innovation fields and is currently used in pharmacology by development-fostering agencies [35,36,37,38,39]. TRLs fit very well in the proposal of a roadmap providing hierarchized development concepts and enabling a step-by-step approach. 

Almost no peer-reviewed publication specifically covering TRLs in pharmaceutical development is available, either for small molecules, HMPs, or biotechnological products. Moreover, most published TRL scales have general concepts and examples, but lack descriptors defining boundaries, leaving room for inconsistent subjective choices that might vary among projects. Such a problem was detected and addressed in the chemical industry [40]. 

To face these challenges, we propose a TRL-guided HMP development roadmap applicable to Brazilian innovations aiming at worldwide marketing. It is organized in a step-by-step set of actions grouped into six domains, defined by clear boundaries, and compliant with international guidelines. 

This proposal applies to HMPs, based on herbal preparations (HPs) as active pharmaceutical ingredients (API), and not to isolated or purified substances. It also applies to plants not included in official monographs, regardless of previous traditional use. For the development of HMPs based on traditional or well-established use, we refer the reader to the following references [41,42,43,44,45,46,47]. Moreover, for the innovations addressed here, previous ethnopharmacological information is helpful, but not mandatory.

## 2. Results and Discussion

Following the original Mankins style [33,48,49], we discuss each technology readiness level (TRL) main attribute, citing examples and providing brief descriptions of associated costs and funding sources. Table 1 outlines the primary deliverables for each level defined by Mankins, alongside our proposed adaptations for herbal medicinal products (HMPs). Additionally, Table 1 provides a visual summary of the key concepts and tasks associated with each TRL as applied to the development of HMPs.

### 2.1. TRLs for Herbal Medicinal Products

#### 2.1.1. **TRL-1**

**TRL-1** was described as “basic science envisioning an application” [33,49,50,51]. For chemicals, it was summarized as a “concept” [40]. BARDA [38] describes it as “active monitoring of scientific knowledge database”. 

For HMPs, the starting point is the identification and reporting of an herbal substance (HS) or herbal preparation’s (HP) pharmacologically relevant effect. This comes either from an ethnopharmacological observation or laboratory experimentation. Both demand envisioning a connection between an effect and a medical need [35]. Examples of these two independent possibilities are: 1- an ethnopharmacological description of a plant infusion with psychic effects, and 2- an experimental finding of vasodilating properties of a plant extract. The associated costs typically involve academic research expenditures, encompassing both experimental work and field-based ethnopharmacological surveys. Funding for this stage is commonly provided by basic science funding agencies.

#### 2.1.2. **TRL-2**

**TRL-2** “formulates the technology concept or application, with no experimental proof or detailed analysis supporting the conjecture” [33,49,52]. The R&D fostering agencies agree on “developing hypotheses and experimental designs” [35,36,37,38]. Therefore, **TRL-2** creates a project focused on a product. 

For HMPs, we propose that **TRL-2** devises a technological application and delivers a proof-of-concept (PoC) research project based on the literature and experimental data. It starts addressing issues on the HS and/or HP and chooses at least one experimental model with translational capability. The literature review must be comprehensive and preferably include some data produced by the responsible research group. This difference from the original NASA concept is due to the higher dependence of pharmaceutical development on previous experimental data to design a reliable PoC. 

The progression of the two examples mentioned at TRL-1 involves the formulation of research projects aimed at testing the antidepressant potential of the plant with psychological effects or the antihypertensive potential of the extract with vasodilating properties. The associated costs typically fall within the scope of small- or medium-sized academic projects sponsored by basic-science funding agencies.

#### 2.1.3. **TRL-3**

**TRL-3** delivers an “analytical and experimental critical function and/or characteristic proof of concept” [33,49,52]. The R&D fostering agencies agree on the “proof of concept”, and BARDA adds identifying a target or candidate and demonstrating relevant in vitro activity [35,36,37,38].

For HMPs, we propose that **TRL-3** performs the R&D fostering agencies (PoC) on a bench scale according to the research plan described in **TLR-2**. The results must justify the progression to **TRL-4** and may demand more than one assay. The first example progresses to the performance of an in vitro test for serotonin reuptake and/or an animal model for depression [53]. The second could be an in vitro vasodilation test and/or an animal model for systemic arterial hypertension [54]. As mentioned above, the associated costs typically fall within the scope of small- or medium-sized academic projects sponsored by basic science funding agencies.

#### 2.1.4. **TRL-4**

**TRL-4** goes further in the validation. It must support the concept formulated earlier and be consistent with the requirements of potential system applications [33,49,52]. Moorhouse also adds “credible for design and performance conditions” [55]. The R&D fostering agencies focus on testing in a defined laboratory or animal model, and BARDA specifically mentions “optimization” [35,36,37,38].

For HMPs, we propose HP optimization on a bench scale and broad validation through several independent assays. **TRL-4** is the most laborious and complex stage up to this point and may involve the work of different groups for years. It holds the first major risk inflection point by testing optimized HPs in the most translational disease models. Here, both examples converge to a chemically defined HP with characterization and quantification of constituents with therapeutic activity or marker compound(s) and several in vitro and in vivo tests. The costs and timeliness rise substantially. The challenges might be faced by academic centers alone, or in collaboration with manufacturers and/or contract research organizations (CROs). The costs might be public-sponsored by large-sized academic research projects and may start involving private resources from investors and manufacturers.

#### 2.1.5. **TRL-5**

**TLR-5** performs “component or prototype validation in a development-relevant environment” [33,34,49,50,52,55]. 

For HMPs, we define “component” as a candidate HP produced through optimized and standardized processes. “Validation in a development-relevant environment” is equated with conducting “Good Laboratory Practice (GLP) non-clinical studies” [35,36,37,38]. GLP testing commences only when the phytochemical profile of the HP candidate is well defined, achieved through standardized preparation procedures and bio-guided studies.

Hence, **TRL-5** encompasses two primary tasks: scaling up from the bench to the pilot scale and conducting GLP animal testing. These activities are usually carried out in specialized facilities, including CROs. Costs associated with **TRL-5** are generally within the same range as those of **TRL-4**. While some public funding sources may still be available, the reliance on investors and manufacturers tends to increase.

#### 2.1.6. **TRL-6**

**TRL-6** refers to a “system/subsystem model or prototype demonstration in a relevant environment (ground or space)” [33,34,49,50,52,55]. 

For both drugs and HMPs, the “relevant environment” corresponds to the “first-in-human” testing in phase I clinical trials conducted under Good Clinical Practices (GCP), typically conducted in a medical facility [35,36,37,38]. Costs escalate significantly due to the manufacture of the candidate HMP for human use and GCP testing. While some public funding sources may still be available, the reliance on investors, manufacturers, and banks further increases.

#### 2.1.7. **TRL-7**

**TRL-7** is the “system prototype demonstration in the expected operational environment” [33,34,49,50,51,52,55]. There is a clear parallel between the “expected operational environment” and the phase II clinical trials conducted with patients with the condition potentially treated by the HMP candidate [35,36,37,38].

This TRL holds the second major risk inflection point because, in the case of success, the risks of failure in further stages are reduced. In case of failure, the project shall be aborted or deeply revised. The costs keep rising. Because the technological risks are still high, some public funding might be expected, along with a higher amount of private capital from investors, manufacturers, and banks.

#### 2.1.8. **TRL-8**

In the TLR-8, the “actual system is completed and qualified through test and demonstration” [33,34,49,50,51,52,55]. For pharmaceutical development, it means that an Investigational New Drug (IND) is reviewed and approved by the regulatory agency.

The parallel with phase III clinical trials is evident. This stage is designed to generate statistically significant evidence of HMP efficacy and safety in large patient populations with the target disease [35,36,37,38]. The HMP used is that intended for marketing authorization. Costs associated with **TRL-8** are the highest of the development process. **TRL-8** alone can incur expenses equivalent to those of **TRLs 1** to **7** combined. However, risks could be lower compared to **TRL-7**. Costs are typically privately sponsored by manufacturers, along with contributions from investors and banks.

#### 2.1.9. **TRL-9**

**TRL-9** is an “actual system flight-proven through successful mission operations” [33,34,49,50,51,52,55]. This was translated by “post-marketing studies and surveillance of the HMP after approval for use” [35,36,37,38]. By definition, all technologies that succeed in being applied in actual systems go eventually to **TRL-9**. 

In certain instances, new formulations may be developed with higher or lower concentrations, slow-release properties, or tailored for pediatric use, necessitating additional clinical trials (phase IV or post-marketing). Despite the potential inclusion of phase IV clinical trials, costs typically decrease substantially and are usually sponsored by the manufacturer.

### 2.2. TRL Domains and Boundaries

Given the complexity of HMP development, we established six technological domains whose progression facilitated the delineation of the boundaries for technology readiness levels (TRLs): herbal substance, herbal preparation, herbal medicinal product, analytical development, non-clinical assays, and clinical trials. Table 2 summarizes the deliverables and main regulations applicable for HMPs at each level.

#### 2.2.1. Herbal Substance

HMPs’ **TRLs 1** and **2** rely on a comprehensive literature review, eventually supplemented by any original data provided by the responsible researcher. Older publications can be valuable if they contain human data. However, these reports may suffer from missing, outdated, or unreliable botanical data, inconsistencies in subspecies, or phenotypical variations. Researchers must diligently address these inconsistencies, striving to include all potentially relevant information while highlighting sources of confusion and detailing how these issues were resolved to ensure reliable botanical identification. Additionally, data on species within the same genus may be useful in predicting the expected phytochemical and biological profiles of the chosen plant [56]. **TRL-2** also involves gathering information on genetic resources and assessments related to traditional knowledge, in compliance with the Nagoya Protocol [29]. 

For **TRL-3** proof of concept (PoC), botanical identification must be conducted by a certified botanist using a voucher sample, ideally including the flower, which should be deposited in an official herbarium. The collection process must be meticulously documented, including GPS location, date, time, and weather conditions (preferably dry) at the time of collection, as well as phenological information and the quantity collected. Records of conservation conditions (light and humidity) are of utmost importance. The quantity stored must be sufficient to facilitate counter-proof tests until the completion of **TRL-4**. Henceforth, the species should not change, except for official naming updates. If there is a change in the species, an impact analysis must be conducted to determine which phytochemical and biological tests need to be repeated.

**TRL-4** needs HS full traceability, following the procedures outlined for **TRL-3**. To optimize conditions, gathering various batches of HSs from different locations and during different seasons is essential. This process is time-consuming and progresses from **TRLs 4** to **7**, making an early start crucial. Each batch must be of sufficient quantity and stored appropriately to facilitate repeated assessments for counter-proof tests until the conclusion of **TRL-7**.

**TRL-4** also involves addressing the HS supply chain. Sustainable collection practices are viable if the plant is abundant in the wild and collection methods do not harm the environment. This approach may even yield environmental benefits by engaging rural populations as conservation agents [57]. Agroecological approaches and organic agriculture offer viable options as well. Strains can be selected, domesticated, and cultivated on a small scale to initiate the development of a cultivar. Plant strains exhibiting the desired metabolite profile and biological activity can be chosen for pilot-scale cultivation, typically encompassing less than one hectare [58,59].

**TRL-5** achieves consistency in production type (collection or cultivation), selects the optimal strain, collection time, and implements effective conservation practices which are paramount for scalability. This stage also initiates the development of Good Agricultural and Collection Practices (GACP) and the qualification of suppliers. Rigorous control of production’s initial stages is imperative, encompassing GACP and Good Herbal Processing Practices (GHPP) [60,61]. From **TRL-5** onwards, the HS must undergo quality controls to ensure plant identification, chemical profiling, and detection of impurities (including other materials and plants). Any deviations in the supply chain or composition need an impact analysis on the product. Actions to increase the production scale commence at this stage, continuing through **TRL-7** or **8**. Initial non-clinical GLP tests can be conducted with pilot batches, while long-term pivotal tests and clinical trials are preferably performed using industrial batches.

At **TRL-6**, the focus shifts to consolidating the supply chain and finalizing composition tests. By **TRL-7** the HS production scales up to industrial batches, ranging from hundreds of kilograms to tons, depending on the equipment used. A qualification master plan becomes necessary for phase II clinical trials [62,63], and Good Agricultural and Collection Practices (GACP) must be implemented to provide HS batches for use in phase II and III clinical trials [61].

At **TRL-8**, the raw materials utilized must be those intended for HMP registration. Proof of compliance with GACP guidelines is essential. While formal GACP certification is not mandatory in Brazil, it is required in the European Community to simplify assessment and provide assurance to regulatory bodies [61]. The FDA mandates compliance with the full GACP standard for an IND PH3 Investigational New Drug, as outlined in 21 CFR 321.23. Certification is only necessary for the New Drug Application phase [64].

#### 2.2.2. Herbal Preparation

**TRL-1** ideally provides some information on the extractive methods and the extract’s composition. At **TRL-2**, a thorough literature review should encompass all available information from scientific and patent databases concerning methodological variations and hypotheses regarding active, toxic, and inert components. Initiating a bio-guided study aimed at isolating and identifying or characterizing constituents with therapeutic activity or markers is advisable at this stage. If conducted, high-throughput and high-content screenings (HTS, HCS) of extracts or fractions commence here and continue throughout **TRL-3** [22,65].

At **TRL-3,** a PoC must use the best possible extract, considering the literature review, the investigators’ capabilities, and the budget available. The quantity produced shall be sufficient for repetitions and comparisons until the conclusion of **TRL-4**. The extractive method must be thoughtfully described and justified. Preferably, the solvents should be compatible with a future industrial scale-up; otherwise, some tests may need to be repeated. HP candidates must undergo chemical characterization to the fullest extent possible with the available resources. The bio-guided study should identify constituents with known therapeutic activity, markers, or at least the active phytochemical classes.

At **TRL-4**, the focus is on optimizing extractive conditions at a bench scale to maximize active components while minimizing inert or potentially toxic ones [66,67]. This includes testing various equipment, solvents, techniques, and drying methods [68,69,70]. The extracts undergo evaluation through a series of biological assays to confirm their pharmacological activity [69,71]. **TRL-4** is considered complete only when the chemical profile of the herbal preparation (HP) candidate is optimized at the bench scale through bio-guided studies. Additionally, attention is given to HP stability. Adequate samples must be stored for repeated testing until **TRL-7** or **8**. The entire process must be thoroughly described, and storage conditions must be traceable and auditable.

At **TRL-5**, the HP candidate production is scaled up to pilot or semi-industrial quantities. The challenge lies in maintaining or improving the characteristics selected in the previous TRLs. The pilot process must be robust, reproducible, and capable of delivering a stable product. This lays the foundation for implementing Good Manufacturing Practices (GMP) for the HP, which are required for regulatory-compliant Good Laboratory Practice (GLP) preclinical studies. Typically, this process is carried out by companies specialized in HP manufacture and should adhere to GMP, or in some countries, be GMP-certified. Development of the extractive method is finalized with at least three pilot batches, each comprising at least five kilograms of dry extract, accompanied by the respective analytical reports [16,47,72].

The industrial batches are developed during **TRL-6** and **TRL-7**. The phases I and II clinical trials can be run with either pilot or industrial batches manufactured in compliance with GMP, with validation and certification processes initiated [38,73]. The **TRL-8** phase III clinical trial must be performed with GMP-certified industrial batches identical to those intended for licensing [74]. Information on batch-to-batch variations and the demonstration that the API (HP) is safe and effective within the tolerated variability is required [74].

##### Herbal Preparation Concepts in Europe and Brazil

In Europe and Brazil, extracts can be classified into three categories, according to the European Pharmacopoeia 11th edition [41,75] and the Brazilian Pharmacopoeia 6th edition [76]: 

Standardized extracts (i), in which constituents with therapeutic activity are known. The amount of native or genuine (without excipients) extract is variable, adjusted within the range defined for the active component—the adjustment is carried out with inert excipients or by blending production batches with a higher or lower content of active constituents, resulting in a variable amount of native HP (e.g., *Atropa belladonna*). Quantified extracts (ii), which are adjusted within a defined range of compounds (active markers), whose relation to the activity is proved through clinical trials, and, therefore, generally accepted to contribute to the therapeutic activity. The amount of native (genuine) extract is constant and the adjustment can only be achieved by blending extract batches with the same specification and based on a constant amount of native extract (e.g., *Ginkgo biloba*). Other extracts (iii) are mainly defined by their manufacturing process (state of the drug to be extracted, solvent, extraction conditions) as well as their specification. There is no adjustment for a constituent or group of constituents since the active substance is the native extract, whose amount is constant. The constituents with known therapeutic activity or markers are not known. Therefore, analytical markers are indicated, and their contents are batch-specific, informative, or recommended to have a minimum content referred to (e.g., *Valeriana officinalis*). In the case of traditional use, the extraction process should not change. 

#### 2.2.3. Herbal Medicinal Product

This domain encompasses administration route, formulation, intellectual property (IP), and partnerships. **TRL-2** and **TRL-3** focus on HP solubility and compatibility with vehicles for both in vitro and in vivo testing. In addition to academic sources, conducting a preliminary review of patent databases is advisable to assess freedom to operate.

At **TRL-4**, the physical form and administration route of the HP are defined, and formulation development commences after gathering information on API solubility and stability [77]. Seeking professional advice on intellectual property is advisable to ensure freedom to operate and explore opportunities for patenting formulations that enhance API delivery and stability. For academic projects, this is an opportune time to explore potential partnerships with manufacturers.

At **TRL-5**, GLP tests are conducted with a formulation as close as possible to that intended for human use, considering animal testing peculiarities. Solid formulations for oral use, such as pills and capsules, cannot be directly administered to animals. Instead, GLP guidelines for oral toxicity studies permit administration via gavage, dissolved in drinking water, or mixed with food [78]. Additionally, Brazilian guidelines allow oral tests to be conducted with the HP [79]. Typically, partnerships with manufacturers are already in operation, and intellectual property (IP) issues have been addressed.

**TRL-6** phase I and **TRL-7** phase II clinical trials must be performed with the HMP manufactured in compliance with GMP and stability tests compatible with the clinical trials’ duration. The investigational product manufacture happens in a GMP-like environment but is not necessarily GMP-certified [38,73]. Still, there might be variations in the dose/concentration of the product that goes to the market. If the claim is based on traditional information, traditional doses and concentrations must be followed. 

At **TRL-8**, phase III clinical trials are conducted with the GMP-certified formulation intended for marketing authorization, ensuring stability throughout the study duration [16,38,73]. If there were differences between the formulation used in phase II and phase III clinical trials, the impact on safety and efficacy must be addressed and the modifications justified. For dossier submission, stability tests must be finalized for at least three HMP batches in their packaging. The Nagoya Protocol mandates notification of the final product’s genetic resources and associated traditional knowledge assessment [29]. 

At **TRL-9**, the HMP is finalized. However, it is feasible to develop new formulations for alternative routes, varying concentrations, slow-release, or pediatric use, which typically require new clinical trials for marketing authorization.

#### 2.2.4. Analytical Development

**TRL-2** proposes parameters for HS and HP characterization. For known HSs, the best sources are official pharmacopoeias or compendia [45,46,62,76,80,81,82,83]. Each country has its list of official legally binding pharmacopoeias. For innovative HS, the pharmacopoeias and scientific publications provide insights on standards to follow. HS characterization typically includes descriptions of the plant part(s) used, purity, chemical content, contaminants, etc. [84,85,86]. The HP candidate usually contains substances or chemical classes associated with therapeutic or toxic effects, which can serve as positive and negative markers for HP optimization and quality control. It is recommended to perform a bio-guided isolation of bioactive compounds, especially when commercial standards are unavailable.

At **TRL-3,** analytical methods must be employed for HS and HP characterization by techniques such as thin-layer chromatography (TLC), high-performance liquid chromatography (HPLC), gas chromatography (GC), and mass spectrometry (MS). A fingerprint chromatogram with all peaks and times, along with the identification of some components, is recommended.

**TRL-4** requires HPs’ comprehensive qualitative and quantitative characterization by different methods produced from several HS batches. Bio-guided identification of active compounds and markers progresses, alongside high-throughput screening (HTS) or high-content screening (HCS), if applicable. The analysis should identify the major chromatographic peaks. Similar objectives can be achieved through MS virtual libraries. Hyphenated methods such as HPLC-PDA, UHPLC-PDA-MS, HS-SPME-GC-MS, or GC-FID/MS are increasingly used [87,88,89]. Knowledge of positive and negative markers should increase through bio-guided studies aimed at compound isolation, especially for species lacking traditional use or literature data. All HPs tested thereafter must have analytical reports in the CTD format. ANVISA and EMA have published guidelines adapted to HMPs [90,91,92].

**TRL-5** needs protocols for HS and HP quality control. The analytical methods must be either pharmacopoeic or independently developed and validated addressing specificity/selectivity, linearity, working range, precision, accuracy, detection/quantification limits, and robustness [81,93,94]. In Brazilian regulations, these methods can replace the phytochemical assays for quality control, when technically justified [95]. 

From **TRL-6** onwards, the requirements expand to include the evaluation of formulations for human use, as well as stability testing. At this stage, active ingredients or markers with known therapeutic effects are identified, and all analytical methods must adhere to pharmacopoeial standards or be thoroughly validated. Additionally, a comprehensive chromatographic profile (fingerprint) is established, alongside stringent analysis of potential contaminants, including pesticides, heavy metals, adulterants, microorganisms, and toxins. These analyses must conform to the regulatory requirements of the target country for licensing, primarily following official pharmacopoeias. For **TRL-8**, stability testing is conducted on products in their final packaging. **TRL-9** uses the methodologies established in previous stages, applying them to every industrial batch. New analytical methods are developed or adapted as necessary to accommodate any new formulations eventually introduced.

#### 2.2.5. Non-Clinical Assays 

The purpose and number of non-clinical assays for innovative HMPs depend on the documented human exposure. In all cases, **TRL-1** relies on some previous data. **TRL-2** performs a comprehensive literature review, including all relevant in vitro and in vivo data on HSs, HPs, and HMPs from the same species. In some cases, data on other species from the same genus may also be helpful. The review embraces decades of information in pharmacopoeias, scientific journals, theses, books, websites, congress abstracts, and others. If available, it is advantageous to include data produced by the development team. 

The PoC experiment characterizes **TRL-3.** It includes in vitro assays of relevant targets and/or in vivo disease models. Appropriated negative and positive controls are mandatory. The assay(s) choice shall be justified, based on the potential for extrapolation to humans, and described in detail, including allometric dose transposal. The report(s) must address data quality and sufficiency to progress to the next level. 

At **TRL-4,** assays increase in quantity, quality, and translational capability. The hypotheses on active compounds and MoA advance, helping efficacy and safety assessments [96]. In vitro, multitarget screening helps to elucidate the MoA and discloses off-target effects [97,98,99]. 

Extracts, fractions, and pure compounds must be tested to identify those participating in the biological effects. The impact of minor and unknown components must be addressed. A set of tests shall be developed for bio-guiding the HP optimization. These should be easy to run (preferably in vitro) and may evolve as knowledge accumulates. Dose–response curves for different HPs and active isolated compounds shall be produced. Confirmatory assays examining the same and other related pathways and disease models in different species are advisable [100,101,102]. Collaboration between groups mastering different methods is encouraged. 

Whenever structural information is available, fast and low-cost in silico tests are also recommended. Its relevance grows as quantitative structure–activity relationship (QSAR) modeling and chemical databases improve [69,103]. Also, some tests integrating responses of various compounds in mixtures are under development [104,105,106]. 

The careful selection, performance, interpretation, and reporting of the best models are of foremost importance. Animal model predictions are often not confirmed in clinical trials [107,108,109]. Therefore, the potential for extrapolating the conclusions to humans shall be reviewed and deepened. Experiments must address aspects of efficacy, toxicity, and capability to reach the target tissues/organs (PK). The report should discuss the experiments’ strengths and weaknesses for translation to human outcomes. This includes the relevance of the experimental models, the allometric dose transposal, and comparisons with the controls. 

The authors recommend performing the “killer experiment” as soon as possible after the PoC, at the end of TRL-3 or the beginning of **TRL-4.** It aims at “killing” an unviable technology before significant resources are spent. Its performance builds trust that, once optimized, the technology will be competitive in the market [110]. 

**TRL-5** focuses on regulatory-demanded GLP animal safety and toxicology tests [78,79,111]. A peculiarity of HMPs is the possibility of waiving some non-clinical tests if sufficient data from other sources are available [112,113]. 

For HMPs with a history of human use, the requirement for GLP toxicology studies is determined by how closely the new HP matches the one previously used by humans. The comparison encompasses composition, clinical application, exposure (dose and duration), and the frequency and severity of any known adverse reactions [16,79]. Nonetheless, genotoxicity and reproductive toxicity, whose risks are difficult to detect clinically should be addressed, but exemptions apply [79,114,115]. The potential interaction with other medicines also needs to be addressed [116,117].

For innovative HMPs, with no prior human exposure, most regulatory agencies demand the same set of GLP toxicological tests required for small molecules, starting at **TRL-5** and finishing at **TRL-7** or **TRL-8** [79,118].

At **TRL-5**, tests needed for phase I clinical trial authorization include cytotoxicity, genotoxicity, local tolerance (if applicable), acute toxicity, short-term repeated dose toxicity, and safety evaluations of the cardiovascular, respiratory, and central nervous systems [16,79,111,119]. 

**TRL-6** encompasses tests necessary for phase II clinical trial authorization, specifically repeated dose toxicity and reproductive toxicity studies [62,111]. Additional tests may be required like the HMP batch-to-batch quality control biological test according to the *United States Pharmacopeia*, chapters 1032 and 1033, required by the FDA [120,121]. Its development starts at **TRL-4** and finishes at **TRL-8.** Another example is the pharmacokinetic (PK) and pharmacodynamic data (PD) demanded by the EMA for standardized HPs, but not for quantified and other HPs. PK and PD data may be difficult to obtain if the effect depends on mixtures but must be obtained in standardized extracts for at least one constituent with known therapeutic activity. Also, the FDA states that “if feasible, chemical constituents of a drug product that contribute to toxicity or pharmacology should be assessed in the pharmacokinetic/toxicokinetic studies” [62]. These PK and PD data are not required for HMPs by most other regulatory agencies [16,79,92]. 

If applicable, **TRL-7** performs the tests demanded for continuous or long-term use such as chronic toxicity. Carcinogenicity is required if there is evidence of genotoxicity, of carcinogenicity in repeated dose toxicity, or if the composition holds a structure related to a known carcinogen [79,111,122]. These must start before the phase III clinical trials and have enough interim data to support the duration of these trials. Complete data will be required for HMP licensing [62].

At TRLs 7 and 8, regulators accept in silico testing for assessing the safety of minor components in standardized extracts. This method is particularly valuable when the molecular structure is known and traditional toxicological in vivo or in vitro tests are impractical [123,124,125]. 

##### Allometric Dose Conversion

Certain biological effects occur in vitro and in vivo at doses exceeding those feasible for human use, making the findings clinically irrelevant. Allometric dose conversion is employed to achieve comparable target tissue concentrations across different species. For an accurate calculation, it is advised to adjust the methodologies used for isolated substances to HMPs [126,127]. 

##### Killer Experiment

The “killer experiment” is aimed at stopping a project with low chances of success before consuming significant resources. If a well-designed and well-conducted killer experiment does not demonstrate the project’s unfeasibility, this is a compelling argument for its continuity [110]. Examples of “killer experiments” adapted for HMPs are:

a. Demonstration of low efficacy in doses suitable for humans after allometric calculation in the gold-standard model of the target disease.
b. Demonstration of efficacy/toxicity balance worse than a comparator under development or already in the market, even after optimization. Note: if the MoA is different from the available comparators, the product may still be viable, even if the potency is lower or the toxicity is higher, because it might work in cases not responsive to the comparator.
c. Demonstration that a component responsible for a large part of the effect does not reach an effective concentration in the target tissue/organ in a dose suitable for humans after formulation optimization.
d. Demonstration of active components’ instability, except for unmet medical needs. For these cases, the search for stable related compounds is advisable.
e. Demonstration of manufacturing costs incompatible with the therapeutic indication.

#### 2.2.6. Clinical Trials

Clinical trials for INDs have robust international regulations, which are out of the scope of this paper [118,128]. The specific demands for HMPs depend on the degree of innovation. In all cases, **TRL-2** performs a comprehensive review of all available sources, looking for traditional use, pharmacovigilance, clinical trials, accidental intoxications, and other reports on human use. Literature-based comparisons with existing drugs for the same indication are advisable to reinforce the medical utility of the proposed innovation. The review must be updated at **TRL-5**, before starting clinical trials. 

For innovative HMPs, with no prior human exposure, most regulatory agencies follow the IND regulations [118]. In cases with reliable data on prior human use, some IND requirements could be exempt. The sponsor is recommended to contact the regulatory authority to obtain advice on the clinical development plan [62]. 

At **TRL-6**, phase I clinical trials address aspects of safety and PK for the constituents with known therapeutic activity. The FDA and EMA have specific recommendations applicable to first-in-human studies which are worth consulting [73,129]. For HMPs, whenever enough information is available from traditional use, phase I clinical trials in normal volunteers are usually waived. A substantial prior human use of the HMP generally conveys confidence that similar amounts are safe for small numbers of carefully monitored subjects in phase II trials [16,62]. However, the FDA recommends getting PK parameters to achieve the same objectives of clinical pharmacology studies for nonbotanical drugs, if the major active constituent(s) in a botanical product is(are) known [62]. 

At **TRL-7**, phase II clinical trials explore efficacy, safety, and dose-finding in small populations. If the phase I clinical trial is waived and phase II is the first-in-human test, consult the FDA and EMA-specific regulations [73,129]. This is one of the most critical stages of pharmaceutical development, often referred to as the “valley of death”, where most clinical failures are seen [97]. Although generally regarded as a PoC in humans, the phase II clinical trials do not necessarily produce statistically significant results. Instead, they provide information suggestive of efficacy that enables the sample size calculation for the confirmatory phase III clinical trials. Moreover, **TRL-7** must generate dose-ranging data. Some regulatory agencies demand dose-ranging studies if the scientific literature does not contain valid data [16]. The FDA demands the dose selection rationale to be based on experimental human PK data, regardless of previous marketing experience [62]. 

At **TRL-8,** phase III clinical trials confirm efficacy and safety in broader patient populations. These trials are designed to yield statistically significant results for predefined variables through predetermined statistical methods (estimands) [130]. They also expand the safety evaluations to larger cohorts to uncover rare adverse events [16,113]. Achieving marketing authorization from at least one regulatory agency signifies the completion of **TRL-8** criteria. Therefore, it is recommended for sponsors to engage with regulatory agencies before initiating any phase III trial [62]. At **TRL-9**, the sponsor may conduct phase IV clinical trials to further elucidate the HMP’s therapeutic profile or to assess new formulations.

##### Special Issues on Clinical Trials of Herbal Medicinal Products

Although considered the gold standard for randomized-controlled clinical trials, the use of a placebo is especially challenging for HMP development [131]. The possibilities for placebos include excipients and other pharmacologically inert ingredients. However, HMPs usually have typical organoleptic properties such as taste, odor, and appearance that might be difficult to mask [132]. A possibility is using other botanical materials with no pharmacological activity. However, concerns about an unsuspected activity might always arise. Another possibility is a double-dummy design that randomizes participants to take either the active treatment “1” and the placebo of the treatment “2”, or the placebo of the treatment “1” and the active treatment “2” [133]. The FDA understands that it might be difficult to select an ideal placebo for HMPs and encourages the sponsor to consult with the agency beforehand [62]. 

Another challenge is the adjuvant treatment of serious conditions to which the FDA recommends the “add-on to standard care versus standard care” [62]. In Brazil, according to the Brazilian National Health Council, the placebo may be used only in cases where no recognized helpful treatment is available. Also, for these cases, the add-on design is recommended.

To minimize different understandings, the WHO published the guideline “*Operational guidance: Information needed to support clinical trials of herbal products*”. This should be reviewed before designing the trials alongside the national regulations of the country where the HMP is intended to be licensed [16].

##### Ethnopharmacological Information, Traditional Use

The research on NPs has historically been guided by ethnopharmacology. The level of information spans from an anecdotal mention of use to long-term, high-quality pharmacovigilance documentation with detailed descriptions of the HP, therapeutic effects, recommended doses, and adverse events. Whenever available, no human data shall be neglected. The FDA, ANVISA, and EMA demand the submission of all information available on prior human exposure [62].

Ethnopharmacology can be a development starter or accelerator. In some cases, it starts a research program to confirm and clarify the pharmacological potential. In other cases, it justifies skipping some non-clinical assays and clinical trials after scaling-up issues have been addressed. If consistent information on HP use by humans is available, it might even be unethical to submit animals or humans to IND-directed studies [112,113]. 

However, handling ethnopharmacological information holds many challenges. The first is species identification. Many historical records only mention common names, which can be attributed to multiple plants across different botanical families, complicating accurate identification. The next challenge is the HP description, including the part of the plant used, extractive methods, route of administration, and doses, among others. Once these challenges are overcome, the value of the ethnopharmacological source is given by the richness and reliability of the information on human effects. Traditional communities may employ terminology vastly different from that of Western medicine, potentially complicating the interpretation of therapeutic effects. However, if clinical manifestations are documented, it may be feasible to translate these into terms recognizable within Western medical frameworks [134]. This can be the case for more consolidated and documented alternative medical systems such as Ayurveda, Traditional Chinese Medicine, Unani, Siddha, and other herbal medicines [113]. 

Moreover, according to the FDA [62], “when the rationale for developing certain botanical drug products is based on prior clinical experience in alternative medical systems e.g., Ayurveda, Traditional Chinese Medicine, Unani, Siddha, and other herbal medicine and pharmacognosy textbooks), the sponsor may propose to incorporate traditional practices into their clinical protocols. For example, patients may be selected or grouped based on alternative medical practice and treated with specific botanical regimens accordingly, or the final dosage form may be prepared by individual patients according to traditional Chinese or Indian methods”. 

## 3. Materials and Methods

The TRLs for HMPs were based on the original NASA concepts from aeronautical development [33,34,48,49,50,51,52,55,135]. A search was conducted on the Web of Science, PubMed, Embase, and Scopus databases using MeSH descriptors and keywords related to technology readiness levels (TRLs) in pharmaceutical development, identifying 360 references. After removing duplicates, 262 articles were analyzed, and 12 were selected for further consideration based on the specific mention of TRL in any biological field [40,136,137,138,139,140,141,142,143,144,145,146]. None specifically addressed TRLs in pharmaceutical development, including small molecules, biotechnological products, and herbal medicinal products (HMPs) for human use. The closest were in chemistry [40], drugs and vaccines for animal health [145], and the bioindustry [146]. Some R&D agencies’ websites have adapted technology readiness level (TRL) concepts to pharmaceutical development, serving as both starting points and foundations upon which we built our concepts for HMPs [35,36,37,38]. Drawing from these sources and our professional experience, we applied technology readiness level (TRL) concepts to herbal medicinal products (HMPs), creating a list of milestones to be reached at each level. These milestones were categorized into six domains and aligned with relevant guidelines from the ANVISA, EMA, FDA, the International Council for Harmonisation of Technical Requirements for Pharmaceuticals for Human Use (ICH), the Organisation for Economic Co-operation and Development (OECD), and the World Health Organization (WHO). Considering the extensive European expertise in HMP regulation, we embraced the terminology established by the EMA. The correspondence of EMA terminology to that of the FDA and ANVISA is in Table 3.

## 4. Concluding Remarks

This TRL-based roadmap is primarily designed to facilitate collaboration among Brazilian scientists, manufacturers, and funding agencies in the development of innovative herbal medicinal products (HMPs) targeting global markets. However, it could also be beneficial for developers in other megadiverse countries looking to navigate similar paths. 

Biodiversity along with ethnopharmacological information are still very underexploited assets from megadiverse countries for drug discovery and development [147,148,149,150]. Most marketed HMPs are based on European plants or traditional Chinese medicine [151,152]. After the Nagoya Protocol, some large pharmaceutical companies have scaled back their bioprospecting activities [1,22,153]. This presents a potential window of opportunity for research institutions in megadiverse countries. 

The challenges are many. The pharma R&D processes are not mature in tropical countries’ manufacturers. HMPs lack regulatory harmonization, and some legal uncertainties from the Nagoya protocol still remain. Also, the HMPs’ R&D is more complex than synthetic small molecules because the HP composition is susceptible to variations in plant strain, place and season of cultivation/collection, and extraction and storage conditions. This roadmap proposal is an effort to overcome some of these challenges. It should be viewed as a general guidance meant to be tailored to each unique development scenario, acknowledging that every case is distinct and has its own set of specific characteristics.

As previously noted, the established TRL scales lack boundary indicators [28,113]. This was the pioneers’ proposal and the main reason for TRLs’ universality and application to many diverse technologies. Nevertheless, this allows the classification of the same project in different TRLs, depending on subjective judgments. To avoid this ambiguity, besides the TRLs transposal, we created a list of indicators organized into six domains that progress with the TRLs. The domains help project management and allow more precise indicators. 

Due to the universality of the TRL concepts, the transposal consistently met the HMP’s R&D milestones. Aeronautical and pharmaceutical developments share some similarities. Both start with material properties, followed by a proposal of application. However, one important difference was the requirement of experimental data at TRL-2 to design the PoC. This was due to the larger dependence of pharmacology on experimentation. TRL-3 was kept as the PoC proposed by Mankins. This crucial step concerns a low-cost experiment (or group of experiments) that needs to be convincing enough to justify the project’s progression.

TRL-4 is very important for the project’s success and its first major risk inflection point. For HMPs, the “validation in laboratory environment” proposed by Mankins was translated as the complex, long, expensive, and laborious bench-scale HP optimization. In our opinion, the lack of innovation in HMPs is mostly due to insufficient optimization. Several projects have progressed unoptimized to GLP testing or even to clinical trials and went back to initial stages or were abandoned afterward, with inconsistent results. Therefore, we equate TRL-4 with the first “valley of death” [154]. It is the gap between the fundamental research and the regulatory-demanded non-clinical and clinical tests that finalize the innovation process. This gap still endures, despite the availability of non-refundable funding specifically directed to innovation, perhaps due to the unawareness of its importance and complexity [155]. 

The killer experiment is a good managerial principle to avoid spending scarce resources on wrong projects. It reduces technological risk and justifies the scaling-up investment. In case of failure, it might be necessary to abort or to improve the HP candidate, meaning that fundamental science is lacking. Therefore, in some cases, it might drive the interruption of the technological aspect to stimulate fundamental science. It should be envisioned early on at TRL-2 and executed at TRL-3 to TRL-4. Every innovative project achieving TRL-4 demands broader pharmacological knowledge. Hereon, the more the project matures and the closer it gets to the market, the more information will be needed to minimize the clinical trials’ risks. These studies are generally carried out in research centers and shall not be confused with the technological project itself but establish a relationship of reciprocal benefits. In this way, while a technological project progresses to higher TRLs, phytochemical and pharmacological scientific studies can feed it with valuable information and receive suggestions that can motivate new research lines.

TRL-5 has two important goals: scaling up and validating the HP. Considering this, the “relevant environment” proposed by Mankins was translated as the GLP condition. It is also time to improve the manufacturing standards to face the clinical trials’ demands.

TRL-6 has another remarkable similarity between aeronautic and pharmacological development—the first flight and the first human test demand substantial preceding validations. Additionally, the object of development is called by Mankins: “component” at TRL-5, “system/subsystem” at TRL-6, “prototype” at TRL-7, and “actual system” at TRL-8. This bears close similarity with the HMP. At TRL-5, the “component” is the HP and, at TRL-6 and TRL-7, the prototype and system/subsystem correspond to the evolving HMP formulation to be administered to humans. At TRL-8, the “actual system” corresponds to the HMP that goes to the market. In either case, models could be considered one-order-of-magnitude approximations for performance [55].

Mankins also stated that “not all technologies undergo TRLs 6 and 7”, but, by definition, all go through TRL-8, which completes the “system development” [52]. For HMPs, those projects with previous human data can skip at least TRL-6, and in the case of previously established doses according to traditional use, TRL-7 could also be skipped, but, by definition, all successful projects must have approval by at least one regulatory authority, which characterizes the TRL-8 completion.

TRL-9 holds an important difference between HMPs and astronautic development. Except for new formulations demanding new clinical trials, no changes to the HMP or manufacturing chain are allowed. 

As a final remark, the TRLs do not provide insight into the uncertainty in pursuing further maturation levels. Therefore, they are not substitutes for individualized expert assessments of each project. Instead, they provide tools to guide the expert assessment. For risk assessment, an additional metric is needed: the R&D degree of difficulty as described elsewhere [34,52]. 

### Future Implications

This TRL-based roadmap was created to enhance HMP development in megadiverse countries with scientific rigor and regulatory compliance. It facilitates streamlined management and resource allocation, fostering trust among scientists, manufacturers, and funding agencies in the planning and execution of HMP development projects. We anticipate that it will promote sustainable practices and catalyze therapeutic breakthroughs from underutilized plant species, thereby increasing the introduction of innovative HMPs. The future application of this roadmap in concrete cases will reveal areas for potential improvements.

## Figures and Tables

**Table 1:** Technology readiness level roadmap according to Mankins [23,34,40] and its adaptation to herbal medicinal product development (HMP).

**TRL-1**	Mankins	Basic principles observed and reported
	HMP	**Pharmacological effect of an herbal preparation observed and reported**
**TRL-2**	Mankins	Technology concept and/or application formulated
	HMP	**Technological application formulated and proof of concept planned**
**TRL-3**	Mankins	Analytical and experimental critical function and/or characteristic proof of concept
	HMP	**Proof of concept of herbal preparation (API candidate)**
**TRL-4**	Mankins	Component and/or breadboard validation in a laboratory environment
	HMP	**Optimization of herbal preparation in bench-scale and non-GLP validation**
**TRL-5**	Mankins	Component and/or breadboard validation in a relevant environment
	HMP	**Optimization of herbal preparation in pilot-scale and GLP validation**
**TRL-6**	Mankins	System/subsystem model or prototype demonstration in a relevant environment (ground or space)
	HMP	**Herbal medicinal product prototype completes phase I clinical trial**
**TRL-7**	Mankins	System prototype demonstration in a space environment
	HMP	**Herbal medicinal product completes phase II clinical trial**
**TRL-8**	Mankins	Actual system completed and “flight qualified” through test and demonstration (ground or space)
	HMP	**Herbal medicinal product completes phase III clinical trial, and the dossier is approved by one regulatory authority**
**TRL-9**	Mankins	Actual system “flight-proven” through successful mission operations
	HMP	**Herbal medicinal product pharmacovigilance and phase IV clinical trials are performed**

**Table 2:** Main deliverables of each TRL for each HMP development domain with main regulations applicable.

	HS	HP	HMP	AD	NCA	CT
**TLR1**	Initial literature review and data collection					
**TLR-2**	Addressing botanical inconsistencies	Comprehensive literature review	Compatibility with vehicles	Initial parameters’ proposal	Comprehensive literature review	Comprehensive literature review
**TLR-3**	Certified botanical identification	Best extract under current conditions	Advance solubility and compatibility issues	Best characterization under current conditions	Optimal PoC under current conditions	
**TLR-4**	Full traceability supply-chain development	Bench-scale optimization and semi-industrial scale initiation	Advances formulation for pre-clinicals	Consistent characterization	Efficacy and safety confirmation and bio-guided HP optimization	Update literature review
**TLR-5**	Supply chain consolidation	Semi-industrial scale optimization	Advance formulation for human use	Validated methods for HP	GLP tests for phase I CT authorization	Update literature review
**TLR-6**	Stability assessment	GMP-compliant batches	GMP-compliant batches	Validated methods for HP and HMP	GLP tests for phase II CT authorization	GCP phase I clinical trial
**TLR-7**	Industrial scale and GACP implementation	GMP-compliant industrial batches	GMP-compliant batches	Validated methods for HP and HMP	GLP tests for long-term use	GCP phase II clinical trials
**TLR-8**	GACP compliance	GMP-compliant industrial batches	GMP-certified batches	Validated methods for HP and HMP	GLP tests for long-term use	GCP phase III clinical trials
**TLR-9**	GACP certified	GMP-compliant * or certified industrial batches	GMP-certified batches	Validated methods for HP and HMP		GCP phase IV trials and pharmaco-vigilance

HS: herbal substance; HP: herbal preparation; HMP: herbal medicinal product; AD: analytical development; NCA: pre-clinical assays; CT: clinical trials; GACP: Good Agricultural and Collection Practices; GLPs: Good Laboratory Practices; GCP: Good Clinical Practices; The Nagoya Protocol applies to the HSs and HPs in different forms in different countries. * The demand for GMP-compliant or GMP-certified HP at TRL-8 varies in different countries.

**Table 3:** Terminology correspondence between EMA, FDA, and ANVISA.

EMA Term [31]	FDA Term [48]	ANVISA Term [63]	EMA Definition
Herbal substance	Botanical drug substance	Droga vegetal	Mainly whole, fragmented, or cut plants, plant parts, algae, fungi, and lichen in an unprocessed, usually dried form, but sometimes fresh. Certain exudates that have not been subjected to a specific treatment are also considered to be herbal substances. Herbal substances are precisely defined by the plant part used and the botanical name according to the binomial system (genus, species, variety, and author) [114].
Herbal preparation	Botanical drug preparation	Derivado vegetal	Preparations are obtained by subjecting herbal substances to treatments such as extraction, distillation, expression, fractionation, purification, concentration, or fermentation. These include comminuted or powdered herbal substances, tinctures, extracts, essential oils, expressed juices, and processed exudates [115].
Herbal medicinal product	Botanical drug product	Fitoterápico	Any medicinal product, exclusively containing as active ingredients one or more herbal substances or one or more herbal preparations, or one or more such herbal substances in combination with one or more such herbal preparations [116].

## Data Availability

The raw data supporting the conclusions of this article will be made available by the authors on request.

## References

[1] Li J.W., Vederas J.C. (2009). Drug discovery and natural products: End of an era or an endless frontier?. Science.

[2] Firn R.D., Jones C.G. (2003). Natural products—A simple model to explain chemical diversity. Nat. Prod. Rep..

[3] Davison E.K., Brimble M.A. (2019). Natural product derived privileged scaffolds in drug discovery. Curr. Opin. Chem. Biol..

[4] Larsson J., Gottfries J., Muresan S., Backlund A. (2007). ChemGPS-NP: Tuned for navigation in biologically relevant chemical space. J. Nat. Prod..

[5] Bruder M., Polo G., Trivella D.B.B. (2020). Natural allosteric modulators and their biological targets: Molecular signatures and mechanisms. Nat. Prod. Rep..

[6] Newman D.J., Cragg G.M. (2020). Natural Products as Sources of New Drugs over the Nearly Four Decades from 01/1981 to 09/2019. J. Nat. Prod..

[7] Drews J. (2000). Drug discovery: A historical perspective. Science.

[8] Efferth T., Koch E. (2011). Complex interactions between phytochemicals. The multi-target therapeutic concept of phytotherapy. Curr. Drug Targets.

[9] Fibigr J., Satinsky D., Solich P. (2018). Current trends in the analysis and quality control of food supplements based on plant extracts. Anal. Chim. Acta.

[10] Koeberle A., Werz O. (2014). Multi-target approach for natural products in inflammation. Drug Discov. Today.

[11] Stasilowicz A., Tomala A., Podolak I., Cielecka-Piontek J. (2021). *Cannabis sativa* L. as a Natural Drug Meeting the Criteria of a Multitarget Approach to Treatment. Int. J. Mol. Sci..

[12] Stockfleth E., Meyer T. (2012). The use of sinecatechins (polyphenon E) ointment for treatment of external genital warts. Expert Opin. Biol. Ther..

[13] Noor F., Tahir Ul Qamar M., Ashfaq U.A., Albutti A., Alwashmi A.S.S., Aljasir M.A. (2022). Network Pharmacology Approach for Medicinal Plants: Review and Assessment. Pharmaceuticals.

[14] Caesar L.K., Cech N.B. (2019). Synergy and antagonism in natural product extracts: When 1 + 1 does not equal 2. Nat. Prod. Rep..

[15] WHO (2013). WHO Traditional Medicine Strategy: 2014–2023.

[16] WHO (2005). Operational Guidance: Information Needed to Support Clinical Trials of Herbal Products.

[17] Liang Z., Hu H., Li J., Yao D., Wang Y., Ung C.O.L. (2021). Advancing the Regulation of Traditional and Complementary Medicine Products: A Comparison of Five Regulatory Systems on Traditional Medicines with a Long History of Use. Evid. Based Complement. Altern. Med..

[18] Jadhav C.A., Vikhe D.N., Jadhav R.S. (2020). Global and domestic market of herbal medicines: A review. Res. J. Sci. Technol..

[19] Grabher C. (2015). A Governança e a Sustentabilidade Do Extrativismo Do Jaborandi Na Amazônia e Transição Para o Cerrado e a Caatinga. Faculdade de Ciências Econômicas. Programa de Pós-Graduação em Desenvolvimento Rural.

[20] WFO (2024). World Flora Online. https://www.worldfloraonline.org/.

[21] Theodoridis S., Drakou E.G., Hickler T., Thines M., Nogues-Bravo D. (2023). Evaluating natural medicinal resources and their exposure to global change. Lancet Planet. Health.

[22] Atanasov A.G., Waltenberger B., Pferschy-Wenzig E.M., Linder T., Wawrosch C., Uhrin P., Temml V., Wang L., Schwaiger S., Heiss E.H. (2015). Discovery and resupply of pharmacologically active plant-derived natural products: A review. Biotechnol. Adv..

[23] Seile B.P., Bareetseng S., Koitsiwe M.T., Aremu A.O. (2022). Indigenous Knowledge on the Uses, Sustainability and Conservation of African Ginger (*Siphonochilus aethiopicus*) among Two Communities in Mpumalanga Province, South Africa. Diversity.

[24] Pasdaran A., Naychov Z., Batovska D., Kerr P., Favre A., Dimitrov V., Aneva I., Hamedi A., Kozuharova E. (2023). Some European *Gentiana* Species Are Used Traditionally to Cure Wounds: Bioactivity and Conservation Issues. Diversity.

[25] Balkrishna A., Singh R., Gohel V., Arora S., Dev R., Bhattacharya K., Varshney A. (2022). Enteric-Coated Cologrit Tablet Exhibit Robust Anti-Inflammatory Response in Ulcerative Colitis-like In-Vitro Models by Attuning NFkappaB-Centric Signaling Axis. Pharmaceuticals.

[26] Pan American Health Organization (2024). Country Profiles. https://tcim-globalsummit.bvsalud.org/regulations-and-policies/.

[27] Geck M.S., Cristians S., Berger-Gonzalez M., Casu L., Heinrich M., Leonti M. (2020). Traditional Herbal Medicine in Mesoamerica: Toward Its Evidence Base for Improving Universal Health Coverage. Front. Pharmacol..

[28] Heinrich M., Scotti F., Andrade-Cetto A., Berger-Gonzalez M., Echeverria J., Friso F., Garcia-Cardona F., Hesketh A., Hitziger M., Maake C. (2020). Access and Benefit Sharing Under the Nagoya Protocol-Quo Vadis? Six Latin American Case Studies Assessing Opportunities and Risk. Front. Pharmacol..

[29] Convention on Biological Diversity, United Nations (2011). Nagoya Protocol on Access to Genetic Resources and the Fair and Equitable Sharing of Benefits Arising from Their Utilization to the Convention on Biological Diversity.

[30] Castro Braga F. (2021). Brazilian traditional medicine: Historical basis, features and potentialities for pharmaceutical development. J. Tradit. Chin. Med. Sci..

[31] Presidência da República (2015). Lei No 13.123, de 20 de Maio de 2015.

[32] Knoess W., Wiesner J. (2019). The Globalization of Traditional Medicines: Perspectives Related to the European Union Regulatory Environment. Engineering.

[33] Mankins J.C. (1995). Technology Readiness Level: A White Paper.

[34] Héder M. (2017). From NASA to EU: The evolution of the TRL scale in Public Sector Innovation. Innov. J..

[35] BIRAC 1. Drugs (Including Drug Delivery). BIRAC-TRLs: Technology Readiness Levels by BIRAC Across Areas under Biotechnology 2018. https://www.birac.nic.in/webcontent/birac_trl_doc1_drug_12_09_2018.pdf.

[36] IA (2019). Description of Technology Readiness Levels (TRL). tia.org.za. https://www.tia.org.za/core/uploads/2019/12/TRL-1.pdf.

[37] Notander G. (2020). Technology Readiness Levels—TRL—NASA’s Contribution to Horizon 2020. https://www.wmahsn.org/storage/resources/documents/EIT_Health_KIC_A_guide_to_TRL-EIT_health.pdf.

[38] MedicalCountermeasures.gov (2024). Integrated Technology Readiness Levels (TRLS) for Medical Countermeasure Products (Drugs and Biologics).

[39] European Commission (2019). Horizon 2020 Work Programme 2018–2020.

[40] Buchner G.A., Stepputat K.J., Zimmermann A.W., Schomäcker R. (2019). Specifying Technology Readiness Levels for the Chemical Industry. Ind. Eng. Chem. Res..

[41] European Medicines Agency (2010). Guideline on Declaration of Herbal Substances and Herbal Preparations in Herbal Medicinal Products/Traditional Herbal Medicinal Products.

[42] EMA European Union Monographs and List Entries. Human Regulatory Herbal Products 2022; The Committee on Herbal Medicinal Products (HMPC) Compiles and Assesses Scientific Data on Herbal Substances, Preparations and Combinations with a Focus on Safety and Efficacy. This Work Supports the Harmonisation of the European Market: National Competent Authorities Are Able to Refer to One Unique Set of Information on a Herbal Substance or Preparation When Evaluating Marketing Applications. https://www.ema.europa.eu/en/human-regulatory/herbal-products/european-union-monographs-list-entries.

[43] European Medicines Agency (2024). Herbal Medicinal Products.

[44] European Commission (2009). A Guideline on Summary of Product Characteristics (SmPC) Revision 2.

[45] EMA (2022). Guideline on Specifications: Test Procedures and Acceptance Criteria for Herbal Substances, Herbal Preparations and Herbal Medicinal Products/Traditional Herbal Medicinal Products.

[46] EMA (2022). Guideline on Quality of Herbal Medicinal Products/Traditional Herbal Medicinal Products.

[47] WHO (2018). WHO Guidelines on Good Herbal Processing Practices for Herbal Medicines.

[48] Mankins J.C. (1998). Research & Development Degree of Difficulty (R&D3)—A White Paper.

[49] Mankins J.C. (2002). Approaches to strategic research and technology (R&T) analysis and road mapping. Acta Astronaut..

[50] Mankins J.C. (2009). Technology readiness assessments: A retrospective. Acta Astronaut..

[51] Banke J. (2010). Technology Readiness Levels Demystified. Aeronautics. https://www.nasa.gov/topics/aeronautics/features/trl_demystified.html.

[52] Mankins J.C. (2009). Technology readiness and risk assessments: A new approach. Acta Astronaut..

[53] Hao Y., Ge H., Sun M., Gao Y. (2019). Selecting an Appropriate Animal Model of Depression. Int. J. Mol. Sci..

[54] Lerman L.O., Kurtz T.W., Touyz R.M., Ellison D.H., Chade A.R., Crowley S.D., Mattson D.L., Mullins J.J., Osborn J., Eirin A. (2019). Animal Models of Hypertension: A Scientific Statement From the American Heart Association. Hypertension.

[55] Moorhouse D.J. (2002). Detailed Definitions and Guidance for Application of Technology Readiness Levels. J. Aircr..

[56] Krizkovska B., Kumar R., Rehorova K., Sykora D., Dobiasova S., Kucerova D., Tan M.C., Linis V., Oyong G., Ruml T. (2020). Comparison of Chemical Composition and Biological Activities of Eight *Selaginella* Species. Pharmaceuticals.

[57] Brazbio Partnerships for a Better Wolrd. 2022. Brazbio Was Founded in 2018 as a Joint Venture between Centroflora Group, a Brazilian Company That Operates in the Production and Commercialization of Pharmaceutical Extracts from Natural Ingredients, and Givaudan, a Swiss Company and Global Leader in the Production of Flavors and Fragrances. http://brazbio.com/en/our-company/.

[58] Canter P.H., Thomas H., Ernst E. (2005). Bringing medicinal plants into cultivation: Opportunities and challenges for biotechnology. Trends Biotechnol..

[59] CORDIS (2018). Replacement of Contentious Inputs in Organic Farming Systems. Horizon 2020. https://cordis.europa.eu/article/id/442664-new-agroecological-approaches-to-scale-up-organic-farming.

[60] WHO (2017). Fifty-First Report of the WHO Expert Committee on Specifications for Pharmaceutical Preparations.

[61] WHO (2003). Guidelines on Good Agricultural and Collection Practices (GACP) for Medicinal Plants.

[62] FDA, U.S. Department of Health and Human Services Food and Drug Administration Center for Drug Evaluation and Research (CDER) (2016). Botanical Drug Development Guidance for Industry.

[63] EMA, HMPC (2006). Guideline on Good Agricultural and Collection Practice (GACP) for Starting Materials of Herbal Origin.

[64] FDA (2023). 21CFR312.23.

[65] Maitra U., Stephen C., Ciesla L.M. (2022). Drug discovery from natural products—Old problems and novel solutions for the treatment of neurodegenerative diseases. J. Pharm. Biomed. Anal..

[66] Jităreanu A., Trifan A., Vieriu M., Caba I.-C., Mârțu I., Agoroaei L. (2022). Current Trends in Toxicity Assessment of Herbal Medicines: A Narrative Review. Processes.

[67] Quan N.V., Dang Xuan T., Teschke R. (2020). Potential Hepatotoxins Found in Herbal Medicinal Products: A Systematic Review. Int. J. Mol. Sci..

[68] Ramesh P., Palaniappan A. (2023). Terminalia arjuna, a Cardioprotective Herbal Medicine-Relevancy in the Modern Era of Pharmaceuticals and Green Nanomedicine—A Review. Pharmaceuticals.

[69] Djehiche C., Benzidane N., Djeghim H., Tebboub M., Mokrani E.H., Mebrek S., Messaoudi M., Bensouici C., Alsalme A., Cornu D. (2024). Exploring the Therapeutic Potential of Ammodaucus leucotrichus Seed Extracts: A Multi-Faceted Analysis of Phytochemical Composition, Anti-Inflammatory Efficacy, Predictive Anti-Arthritic Properties, and Molecular Docking Insights. Pharmaceuticals.

[70] Madia V.N., De Angelis M., De Vita D., Messore A., De Leo A., Ialongo D., Tudino V., Saccoliti F., De Chiara G., Garzoli S. (2021). Investigation of Commiphora myrrha (Nees) Engl. Oil and Its Main Components for Antiviral Activity. Pharmaceuticals.

[71] Gaurav H., Yadav D., Maurya A., Yadav H., Yadav R., Shukla A.C., Sharma M., Gupta V.K., Palazon J. (2023). Biodiversity, Biochemical Profiling, and Pharmaco-Commercial Applications of Withania somnifera: A Review. Molecules.

[72] WHO (2007). WHO Guidelines on Good Manufacturing Practices for (GMP) for Herbal Medicines.

[73] FDA, U.S. Department of Health and Human Services Food and Drug Administration Center for Drug Evaluation and Research (CDER) Center for Biologics Evaluation and Research (CBER) Office of Regulatory Affairs (ORA) (2008). Guidance for Industry CGMP for Phase 1 Investigational Drugs Administration.

[74] Bodeker G., Ong C.-K., Grundy C., Burford G., Shein K. (2005). WHO Global Atlas of Traditional, Complementary and Alternative Medicine.

[75] Council of Europe, European Directorate for the Quality of Medicines & HealthCare (2023). European Pharmacopoeia (Ph. Eur.).

[76] ANVISA (2022). Farmacopéia Brasileira. Volume 2 Plantas Medicinais.

[77] Nicolai M., Mota J., Fernandes A.S., Pereira F., Pereira P., Reis C.P., Robles Velasco M.V., Baby A.R., Rosado C., Rijo P. (2020). Assessment of the Potential Skin Application of *Plectranthus ecklonii* Benth. Pharmaceuticals.

[78] OECD (2002). OECD Test No. 423: Acute Oral Toxicity—Acute Toxic Class Method.

[79] ANVISA (2019). Estudos Não Clinicos Necessários ao Desenvolvimento de Medicamentos Fitoterápicos.

[80] WHO (2011). Quality Control Methods for Herbal Materials.

[81] USP, United States Pharmacopeial Convention (2024). Herbal Medicines Compendium.

[82] AOACInternational (2023). Official Methods of Analysis.

[83] Medicines & Healthcare Products Regulatory Agency (2024). The British Pharmacopoeia Quality Standards.

[84] Choudhary N., Sekhon B.S. (2011). An overview of advances in the standardization of herbal drugs. J. Pharm. Educ. Res..

[85] Fitzgerald M., Heinrich M., Booker A. (2019). Medicinal Plant Analysis: A Historical and Regional Discussion of Emergent Complex Techniques. Front. Pharmacol..

[86] Muyumba N.W., Mutombo S.C., Sheridan H., Nachtergael A., Duez P. (2021). Quality control of herbal drugs and preparations: The methods of analysis, their relevance and applications. Talanta Open.

[87] Kim M.K., Park S.C., Park G., Choi E., Ji Y., Jang Y.P. (2021). Analytical quality by design methodology for botanical raw material analysis: A case study of flavonoids in Genkwa Flos. Sci. Rep..

[88] Indrayanto G. (2018). Recent Development of Quality Control Methods for Herbal Derived Drug Preparations. Nat. Prod. Commun..

[89] Huang S., Chen J., Li W., Song S., Li X., Yu H., Long F., Chen R., Bao X., Chan K. (2023). Comparison of Volatile Compositions among Four Related Ligusticum chuanxiong Herbs by HS-SPME-GC-MS. Processes.

[90] ICH (2003). M4: The Common Technical Document. https://www.ich.org/page/ctd.

[91] ANVISA (2019). Guia Para Organização do Documento Técnico Comum (CTD) Para o Registro e Pós-Registro de Medicamentos.

[92] EMA (2016). Guideline on the Use of the CTD Format in the Preparation of a Registration Application for Traditional Herbal Medicinal Products.

[93] Agência Nacional de Vigilância Sanitária—Anvisa (2017). RDC 166/2017 Estabelece Critérios Para a Validaçãode Métodos Analíticos.

[94] Sarma N., Upton R., Rose U., Guo D.A., Marles R., Khan I., Giancaspro G. (2023). Pharmacopeial Standards for the Quality Control of Botanical Dietary Supplements in the United States. J. Diet. Suppl..

[95] ANVISA (2014). Resolução da Diretoria Colegiada—RDC N° 26, in Dispõe Sobre o Registro de Medicamentos Fitoterápicos e o Registro e a Notificação de Produtos Tradicionais Fitoterápicos.

[96] Borgonetti V., Les F., Lopez V., Galeotti N. (2020). Attenuation of Anxiety-like Behavior by *Helichrysum stoechas* (L.) Moench Methanolic Extract through Up-Regulation of ERK Signaling Pathways in Noradrenergic Neurons. Pharmaceuticals.

[97] Zurdo J. (2013). Developability assessment as an early de-risking tool for biopharmaceutical development. Pharm. Bioprocess..

[98] Lin A., Giuliano C.J., Palladino A., John K.M., Abramowicz C., Yuan M.L., Sausville E.L., Lukow D.A., Liu L., Chait A.R. (2019). Off-target toxicity is a common mechanism of action of cancer drugs undergoing clinical trials. Sci. Transl. Med..

[99] Tuohongerbieke A., Wang H., Wu J., Wang Z., Dong T., Huang Y., Zhu D., Sun D., Tsim K.W.K. (2024). Xiao Cheng Qi Decoction, an Ancient Chinese Herbal Mixture, Relieves Loperamide-Induced Slow-Transit Constipation in Mice: An Action Mediated by Gut Microbiota. Pharmaceuticals.

[100] Parish T. (2020). In vitro drug discovery models for Mycobacterium tuberculosis relevant for host infection. Expert Opin. Drug Discov..

[101] Sinha S., Vohora D. (2018). Drug Discovery and Development. Pharmaceutical Medicine and Translational Clinical Research.

[102] Franco C.H., Alcantara L.M., Chatelain E., Freitas-Junior L., Moraes C.B. (2019). Drug Discovery for Chagas Disease: Impact of Different Host Cell Lines on Assay Performance and Hit Compound Selection. Trop. Med. Infect. Dis..

[103] Mokhtar F.A., Selim N.M., Elhawary S.S., Abd El Hadi S.R., Hetta M.H., Albalawi M.A., Shati A.A., Alfaifi M.Y., Elbehairi S.E.I., Fahmy L.I. (2022). Green Biosynthesis of Silver Nanoparticles Using Annona glabra and Annona squamosa Extracts with Antimicrobial, Anticancer, Apoptosis Potentials, Assisted by In Silico Modeling, and Metabolic Profiling. Pharmaceuticals.

[104] Pereira F., Aires-de-Sousa J. (2018). Computational Methodologies in the Exploration of Marine Natural Product Leads. Mar. Drugs.

[105] Loiodice S., Nogueira da Costa A., Atienzar F. (2019). Current trends in in silico, in vitro toxicology, and safety biomarkers in early drug development. Drug Chem. Toxicol..

[106] Rim K.T. (2020). In silico prediction of toxicity and its applications for chemicals at work. Toxicol. Environ. Health Sci..

[107] Van Norman G.A. (2019). Limitations of Animal Studies for Predicting Toxicity in Clinical Trials: Is it Time to Rethink Our Current Approach?. JACC Basic Transl. Sci..

[108] Robinson N.B., Krieger K., Khan F.M., Huffman W., Chang M., Naik A., Yongle R., Hameed I., Krieger K., Girardi L.N. (2019). The current state of animal models in research: A review. Int. J. Surg..

[109] Laman J.D., Kooistra S.M., Clausen B.E. (2017). Reproducibility Issues: Avoiding Pitfalls in Animal Inflammation Models. Methods Mol. Biol..

[110] Owens P.K., Raddad E., Miller J.W., Stille J.R., Olovich K.G., Smith N.V., Jones R.S., Scherer J.C. (2015). A decade of innovation in pharmaceutical R&D: The Chorus model. Nat. Rev. Drug Discov..

[111] ICH (2009). Guidance on Nonclinical Safety Studies for the Conduct of Human Clinical Trials and Marketing Authorization for Pharmaceuticals M3(R2) Current Step 4 Version.

[112] (2004). Directive 2004/24/EC of the European Parliament and of the Council. Off. J. Eur. Union.

[113] WHO (2000). General Guidelines for Methodologies on Research and Evaluation of Traditional Medicine.

[114] EMA (2018). Guideline on Non-Clinical Documentation in Applications for Marketing Authorisation/Registration of Wellestablished and Traditional Herbal Medicinal Products.

[115] da Rocha C.F., Flexa C.N.N., de Souza G.C., Pereira A.C.M., Carvalho H.O., do Nascimento A.L., de Jesus Vasconcelos N.J.P., da Silva H.R., Carvalho J.C.T. (2022). Acute and Reproductive Toxicity Evaluation of Ormona((R)) SI and Ormona((R)) RC-Two New Nutraceuticals with Geranylgeraniol, Tocotrienols, Anthocyanins, and Isoflavones-In Adult Zebrafish. Pharmaceuticals.

[116] WHO (2021). Key Technical Issues of Herbal Medicines with Reference to Interaction with Other Medicines.

[117] Mazzari A.L., Milton F., Frangos S., Carvalho A.C., Silveira D., de Assis Rocha Neves F., Prieto J.M. (2016). In vitro Effects of Four Native Brazilian Medicinal Plants in CYP3A4 mRNA Gene Expression, Glutathione Levels, and P-Glycoprotein Activity. Front. Pharmacol..

[118] FDA (2018). The Drug Development Process. https://www.fda.gov/patients/learn-about-drug-and-device-approvals/drug-development-process.

[119] Steinmetz K.L., Spack E.G. (2009). The basics of preclinical drug development for neurodegenerative disease indications. BMC Neurol..

[120] USP (2022). <1033> Biological Assay Validation.

[121] USP (2022). <1032> Design and Development of Biological Assays.

[122] OECD (2009). OECD Test Guideline 451: Carcinogenicity Studies.

[123] Kovarich S., Cappelli C.I. (2022). Use of In Silico Methods for Regulatory Toxicological Assessment of Pharmaceutical Impurities. Methods Mol. Biol..

[124] Little J.G., Marsman D.S., Baker T.R., Mahony C. (2017). In silico approach to safety of botanical dietary supplement ingredients utilizing constituent-level characterization. Food Chem. Toxicol..

[125] Myatt G.J., Ahlberg E., Akahori Y., Allen D., Amberg A., Anger L.T., Aptula A., Auerbach S., Beilke L., Bellion P. (2018). In silico toxicology protocols. Regul. Toxicol. Pharmacol..

[126] Nair A., Morsy M.A., Jacob S. (2018). Dose translation between laboratory animals and human in preclinical and clinical phases of drug development. Drug Dev. Res..

[127] Peters S.A., Petersson C., Blaukat A., Halle J.P., Dolgos H. (2020). Prediction of active human dose: Learnings from 20 years of Merck KGaA experience, illustrated by case studies. Drug Discov. Today.

[128] ICH (2016). ICH: E6 (R2): Guideline for Good Clinical Practice—Step 5.

[129] EMA (2017). Guideline on Strategies to Identify and Mitigate Risks for First-in-Human and Early Clinical Trials with Investigational Medicinal Products.

[130] ICH, ICH Expert Working Group (2019). Addendum on Estimands and Sensitivity Analysis in Clinical Trials to the Guideline on Statistical Principles for Clinical Trials E9(R1). https://database.ich.org/sites/default/files/E9-R1_Step4_Guideline_2019_1203.pdf.

[131] Cañigueral S., Tschopp R., Ambrosetti L., Vignutelli A., Scaglione F., Petrini O. (2012). The Development of Herbal Medicinal Products. Pharm. Med..

[132] Zhang X., Tian R., Zhao C., Tang X., Lu A., Bian Z. (2019). Placebo design in WHO-registered trials of Chinese herbal medicine need improvements. BMC Complement. Altern. Med..

[133] Raus K., Pleschka S., Klein P., Schoop R., Fisher P. (2015). Effect of an Echinacea-Based Hot Drink Versus Oseltamivir in Influenza Treatment: A Randomized, Double-Blind, Double-Dummy, Multicenter, Noninferiority Clinical Trial. Curr. Ther. Res. Clin. Exp..

[134] Pagani E., Santos J.F.L., Rodrigues E. (2017). Culture-Bound Syndromes of a Brazilian Amazon Riverine population: Tentative correspondence between traditional and conventional medicine terms and possible ethnopharmacological implications. J. Ethnopharmacol..

[135] Tomaschek K., Olechowski A., Eppinger S., Joglekar N. (2016). A Survey of Technology Readiness Level Users. INCOSE Int. Symp..

[136] Sutherland I., Ignatova S., Hewitson P., Janaway L., Wood P., Edwards N., Harris G., Guzlek H., Keay D., Freebairn K. (2011). Scalable Technology for the Extraction of Pharmaceutics (STEP): The transition from academic knowhow to industrial reality. J. Chromatogr. A.

[137] Wu C., Wang B., Zhang C., Wysk R.A., Chen Y.W. (2017). Bioprinting: An assessment based on manufacturing readiness levels. Crit. Rev. Biotechnol..

[138] Lakkireddy H.R., Bazile D.V. (2019). Nano-carriers for drug routeing—Towards a new era. J. Drug Target..

[139] Allwardt V., Ainscough A.J., Viswanathan P., Sherrod S.D., McLean J.A., Haddrick M., Pensabene V. (2020). Translational Roadmap for the Organs-on-a-Chip Industry toward Broad Adoption. Bioengineering.

[140] de Araújo e Silva R., Santa Brígida A.I., de Freitas Rosa M., da Silva Neto R.M., Spinosa W.A., Benício de Sá Filho E., Brito de Figueirêdo M.C. (2020). An approach for implementing ecodesign at early research stage: A case study of bacterial cellulose production. J. Clean. Prod..

[141] Daniotti S., Re I. (2021). Marine Biotechnology: Challenges and Development Market Trends for the Enhancement of Biotic Resources in Industrial Pharmaceutical and Food Applications. A Statistical Analysis of Scientific Literature and Business Models. Mar. Drugs.

[142] Pfeifer K., Ergal I., Koller M., Basen M., Schuster B., Rittmann S.K.R. (2021). Archaea Biotechnology. Biotechnol. Adv..

[143] Jenie R.P., Suryana Y., Pambudi S., Widayanti T., Irzaman I., Nurdin N.M., Dahrul M., Iskandar J., Kurniawan A., Siskandar R. (2021). General protocol for ethical conforming development for non-invasive blood biomarker measurement optical device. AIP Conf. Proc..

[144] Becker C.R., Majeed A., Crispin A., Knez A., Schoepf U.J., Boekstegers P., Steinbeck G., Reiser M.F. (2005). CT measurement of coronary calcium mass: Impact on global cardiac risk assessment. Eur. Radiol..

[145] Arnouts S., Brown S., de Arriba M.L., Donabedian M., Charlier J. (2022). Technology Readiness Levels for vaccine and drug development in animal health: From discovery to life cycle management. Front. Vet. Sci..

[146] Smanski M.J., Aristidou A., Carruth R., Erickson J., Gordon M., Kedia S.B., Lee K.H., Prather D., Schiel J.E., Schultheisz H. (2022). Bioindustrial manufacturing readiness levels (BioMRLs) as a shared framework for measuring and communicating the maturity of bioproduct manufacturing processes. J. Ind. Microbiol. Biotechnol..

[147] Myers N., Mittermeier R.A., Mittermeier C.G., da Fonseca G.A., Kent J. (2000). Biodiversity hotspots for conservation priorities. Nature.

[148] Braga F.C. (2021). Paving New Roads Towards Biodiversity-Based Drug Development in Brazil: Lessons from the Past and Future Perspectives. Rev. Bras. Farmacogn..

[149] Yuan H., Ma Q., Ye L., Piao G. (2016). The Traditional Medicine and Modern Medicine from Natural Products. Molecules.

[150] Kim J.K., Kim K.H., Shin Y.C., Jang B.H., Ko S.G. (2020). Utilization of traditional medicine in primary health care in low- and middle-income countries: A systematic review. Health Policy Plan..

[151] Bilia A.R., Costa M.D.C. (2021). Medicinal plants and their preparations in the European market: Why has the harmonization failed? The cases of St. John’s wort, valerian, ginkgo, ginseng, and green tea. Phytomedicine.

[152] Wang M., Franz G. (2015). The role of the European Pharmacopoeia (Ph Eur) in Quality Control of Traditional Chinese Herbal Medicine in European Member States. World J. Tradit. Chin. Med..

[153] Franzen S.R., Chandler C., Lang T. (2017). Health research capacity development in low and middle income countries: Reality or rhetoric? A systematic meta-narrative review of the qualitative literature. BMJ Open.

[154] Calza F., Ferretti M., Panetti E., Parmentola A. (2020). Moving drug discoveries beyond the valley of death: The role of innovation ecosystems. Eur. J. Innov. Manag..

[155] Trivella D.B.B., Bruder M.C.P., Oliveira F.C.B., Porcaro R., Rustiguel J.K., Ribeiro L.B., Felicio R.d., Cunha M.G.d., Nascimento A.F.Z., Zeri A.C.M. (2022). Descoberta de fármacos a partir de produtos naturais e a abordagem Molecular Power House (MPH). Rev. Fitos.

